# A Case Report on Pearson Syndrome With Emphasis on Genetic Screening in Patients Presenting With Sideroblastic Anemia and Lactic Acidosis

**DOI:** 10.7759/cureus.33963

**Published:** 2023-01-19

**Authors:** Celeste Shoeleh, Umberto M Donato, Andrew Galligan, Julie Vitko

**Affiliations:** 1 Pediatric Hematology Oncology, University of South Florida Morsani College of Medicine, Tampa, USA; 2 Health Outcomes and Behavior Lab, Moffitt Cancer Center, Tampa, USA; 3 Pediatric Oncology, Tampa General Hospital, Tampa, USA; 4 Radiology, Moffitt Cancer Center, Tampa, USA; 5 Pediatric Oncology, Moffitt Cancer Center, Tampa, USA; 6 Pathology and Cell Biology, University of South Florida Morsani College of Medicine, Tampa, USA

**Keywords:** mitochondrial disorders, genetic screening, anemia, sideroblastic anemia, “pancytopenia”

## Abstract

Pearson marrow-pancreas syndrome is a rare multisystem mitochondrial disease that is a result of defective oxidative phosphorylation caused by mitochondrial DNA mutations. The average prognosis of infants diagnosed with this disease is death within four years of age. The disease often carries an atypical presentation during the neonatal period causing this rare syndrome to be frequently misdiagnosed. The current report details the diagnosis of Pearson syndrome in a three-month-old male with a history of pancytopenia.

## Introduction

Pearson marrow-pancreas syndrome (referred to as Pearson syndrome) is a rare disease with an estimated incidence of one disease diagnosis/million newborns [1]. Pearson syndrome is caused by single mitochondrial DNA (mtDNA) deletions that affect oxidative phosphorylation. Due to the heteroplasmy phenomenon in mitochondrial diseases, single mtDNA deletions do not have a uniform tissue distribution, resulting in various phenotypes [2]. Pearson syndrome leads to pancytopenia, which manifests as anemia, neutropenia, and thrombocytopenia. Affected infants often show a failure to thrive, difficulty feeding, pallor, exocrine pancreatic insufficiency, lactic acidemia, and microcephaly. Pearson syndrome has a poor prognosis, with infants developing transfusion-dependent anemia and succumbing to metabolic acidosis and/or infections [3,4]. Children who survive develop Kearns-Sayre syndrome (KSS) - a rare multisystemic disorder that reduces the quality of life and can lead to sudden death [3].

Due to the nature of Pearson syndrome, it is diagnosed via genetic testing of mitochondrial DNA (mtDNA). Other clues pointing to a Pearson syndrome diagnosis include sideroblasts on bone marrow evaluation, fecal elastase testing to reveal pancreatic insufficiency, and 3-methylglutaconic acid in urine [5]. However, the phenotypic variability and rare occurrence of this disease result in underdiagnosis. 

In this report, we describe a three-month-old male with Pearson syndrome who presented with a one-week history of vomiting after feeds and irritability. As an example of phenotypic variability of Pearson syndrome, this patient did not experience failure to thrive, exocrine pancreatic insufficiency (according to normal fecal elastase testing), or have evidence of 3-methylglutaconic acid in his urine. This patient did have feeding difficulties, pallor, lactic acidemia, sideroblasts on bone marrow evaluation, microcephaly, and deletion analysis of the mitochondrial genome that revealed a heteroplasmic 5kb deletion in mtDNA.

## Case presentation

The patient was a three-month-old male born prematurely at 33 weeks gestation who presented with a one-week history of vomiting after feedings and irritability. He was admitted due to concerns for severe anemia. His mother stopped feeding Poly-Vi-Sol with iron two weeks prior due to concerns that it was hard on the patient’s stomach. The patient’s medical history included pancytopenia, two blood transfusions due to low hemoglobin levels, microcephaly, and notable dysmorphic features upon birth, including small appearing ears, up-slanting palpebral fissures, flat nasal bridge, and widely spaced nipples. Due to the early blood transfusions, no metabolic screening was conducted. The patient’s mother reported no other health concerns, including bleeding or recent illnesses, and the patient’s growth was within normal limits.

Upon admission, the patient’s physical exam revealed a cough, pallor, dysmorphic features, pale mucous membranes, a systolic murmur, hydrocele, and a significantly delayed capillary refill test. Laboratory values were evaluated and showed reduced red blood cells of 0.53 x 10^6^/uL (normal range: 3.8-5.4 x 10^6^/uL), hemoglobin of 1.6g/dL (normal range: 11.1-14.1 g/dL), and platelets of 14 g/dL (normal range: 32.7-37.3 g/dL) (Table 1). White blood cells of 4.88 x 10^3^/uL (normal range: 6.0-17.5 10^3^/uL) and neutrophil percentage of 3% (normal range: 18-46%) were also reduced. The mean corpuscular volume of 92.5 fL (normal range: 68-84 fL) and lactate levels of 2.7 mmol/L (normal range: 0.5-2.2) were all increased. Increased LDH of 544 U/L (normal 125-220 U/L) was concerning for neonatal leukemia. However, uric acid levels were low at 2.8 mg/dL (normal 3.5-7.2 mg/dL), and a leukemia/lymphoma panel by flow cytometry did not reveal any markers.

**Table 1:** Patient's Laboratory Values at Admission and After Multiple Blood Transfusions

	Values at presentation	Values at discharge post-transfusions	Reference values
Red Blood Cell (10*6/uL)	0.53	3.86	3.8-5.4
Hemoglobin (g/dL)	1.6	12.1	11.1-14.1
Mean Corpuscular Volume (fL)	92.5	85.2	68-84
Platelet Count (g/dL)	14	37	32.7-37.3
White Blood Cell (10*3/uL)	4.88	3.87	6.0-17.5
Neutrophils (%)	3	5	18-46
Reticulocyte (%)	0.13	1.05	0.6-1.6
Immature Reticulocyte Fraction (%)	40	1.6	2.3-15.9
Absolute Reticulocyte	0.0006	0.0405	0.03-0.09
Fetal Hemoglobin (HbF) (%)	17.1	-	40.0-85.0
Lactate (mmol/L)	-	2.7	0.5-2.2
LDH (U/L)	-	544	125-220
Uric Acid (mg/dL)	-	2.8	3.5-7.2

**Figure 1:**
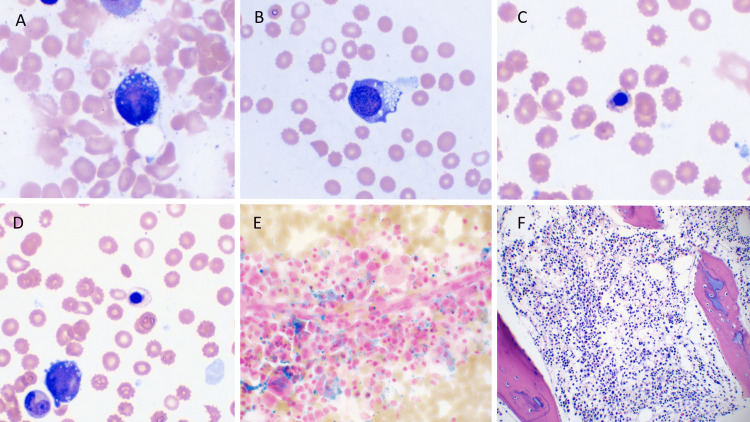
Images of Patient's Bone Marrow Biopsy Images of the Wright's stain bone marrow aspirate smears show the typical abnormalities seen in Pearson’s syndrome characterized by cytoplasmic vacuolization and megaloblastoid changes of erythroid precursors (A, B) and frequent basophilic stippling and abnormal hemoglobinization of orthochromatic normoblasts (C, D). Prussian blue iron stain highlights increased storage iron with ringed sideroblasts (E), also typically present in this subtype of congenital sideroblastic anemia. Image F shows an H&E section of the core biopsy with slightly decreased marrow cellularity, which can progress to aplastic anemia, a late complication of this disease.

Bone marrow biopsy revealed ringed sideroblasts, cytoplasmic vacuolization, dysmyelopoiesis, dyserythropoiesis, and 60% cellularity (normal range: 50%-70%), concerning mitochondrial diseases like Pearson syndrome (Figure 1). However, fecal pancreatic elastase testing (for Pearson syndrome) was normal, and 3-methylglutaconic acid, a possible biomarker for Pearson syndrome, was not found in the patient's urine. 

Three blood transfusions with 10 mL/kg of packed RBCs (pRBC) were conducted to raise hemoglobin levels from 1.6 g/dL to 12.1 g/dL. The patient was also started on Poly-Vi-Sol with iron to further reduce possible nutrition deficiency. Once the patient was stabilized, he was discharged to await results from genetic testing. Deletion analysis of the mitochondrial genome revealed an 80% heteroplasmic 5kb known as m.8483_13459del4977, and a diagnosis of Pearson syndrome was concluded. The deletion affected mitochondrial genes MT-ATP8, MT-ATP6, MT-CO3, MT-TG, MT-ND3, MT-TR, MT-ND4L, MT-ND4, MT-TH, MT-TS2, MT-TL2, MT-ND. 

## Discussion

Pearson syndrome is a rare disease, and due to the variability in symptoms, diagnosis is often delayed or missed. Currently, there are approximately 100 reported cases of this mitochondrial disease [6]. The rarity of this disease, along with the inconsistency of its symptomatic presentation, make it a difficult pathology to diagnose. Although morphological findings are useful in narrowing the differentials, genetic testing is key in diagnosing Pearson syndrome as it identifies the presence of mitochondrial deletions in infants with anemia of an uncertain etiology. 

For example, in this patient, both bone marrow biopsy and the presence of cytopenias of all lineages pointed to a diagnosis of Pearson syndrome. The bone marrow findings included dyserythropoiesis, dysmyelopoiesis with left shift, cytoplasmic vacuolization and megaloblastoid changes of erythroid precursors, basophilic stippling, and increased storage iron with ringed sideroblasts. However, the findings were also indicative of the other differential diagnoses, including both constitutional and acquired disorders. Specifically, copper deficiency, zinc toxicity, congenital megaloblastic anemia, and Fanconi anemia were also considered. Genetic testing identified heteroplasmic 5kb deletions of varying lengths in the mitochondrial genome, including mitochondrial nucleotides 8483 to 13,459. Together, the genetic findings, along with the bone marrow biopsy were extremely consistent with Pearson syndrome presentations found in the literature [7,8]. The inability to explicitly diagnose Pearson syndrome through bone marrow biopsy, and laboratory data underscores the importance of genetic molecular analysis in providing a prompt diagnosis of the pathology.

Standard therapy is mainly supportive with treatment of metabolic acidosis, packed cell transfusions, and granulocyte colony-stimulating factor [9]. The prognosis for Pearson syndrome is still grave, with most infants expiring within the first four years of life due to sepsis or persistent metabolic acidosis - emphasizing a need to find adequate treatments, including cell therapy. Furthermore, the value of an adequate diagnosis of Pearson syndrome in infants is also highlighted by the possibility of phenotypic progression to Kearns-Sayre Syndrome (KSS) in children who survive an early Pearson syndrome presentation. KSS typically presents with ataxia, cardiac conduction defects, ophthalmoplegia, and sensorineural hearing loss [10]. These symptoms can be corrected via hematopoietic stem cell transplantations, but this treatment can often lead to undesired side effects, including renal tubular dysfunction and encephalopathy [4]. This possibility stresses the importance of follow-up care in patients who manage to resolve the symptomatic presentations of the disease.

## Conclusions

This study reports the diagnosis of Pearson syndrome in a patient with an acute history of vomiting after feeds and irritability. Laboratory data and bone marrow biopsy of this infant pointed to a diagnosis of Pearson syndrome, but other possible differentials included Fanconi anemia, congenital megaloblastic anemia, and acquired disorders. mtDNA genetic testing was key in diagnosing this patient as it revealed a heteroplasmic 5kb deletion. 

Pearson syndrome often presents atypically, making it increasingly difficult to distinguish between neuromuscular, infectious and/or digestive pathologies, hence the wide range of possible diagnoses. Due to the variability in the presentation of Pearson syndrome, as witnessed in this case, and its often fatal progression, mtDNA genetic testing should be considered when a patient presents with lactic acidemia, hematologic abnormalities, and features of a progressive disease. Prompt identification may help provide conservative management to the affected patients in the early disease state and improve the clinical course to prevent its phenotypic progression to Kearns-Sayre syndrome (KSS). This case report is important in building on the limited literature pertinent to the clinical intricacies of this rare and harmful disease. Sideroblastic anemia, thrombocytopenia, and/or neutropenia, along with the presence of lactic acidosis, is a pivotal finding that should be thoroughly investigated, and Pearson syndrome should be considered despite varied clinical presentation.
